# Letter to the editor: Difficult removal of exposed peripheral nerve stimulator leads: a report of 2 cases

**DOI:** 10.1097/PR9.0000000000000994

**Published:** 2022-04-05

**Authors:** Harnek S. Bajaj, Denise D. Lester, Robert J. Trainer

**Affiliations:** a Department of Physical Medicine and Rehabilitation, Pain Medicine, Virginia Commonwealth University Health System, Richmond, VA, USA; b Department of Physical Medicine and Rehabilitation, Hunter Holmes McGuire Veterans Affairs Medical Center, Richmond, VA, USA; c Interventional Pain Clinic, Hunter Holmes McGuire Veterans Affairs Medical Center, Richmond, VA, USA

## Letter To Editor:

We have read with interest the recent case report article titled “Difficult removal of exposed peripheral nerve stimulator leads: a report of 2 cases” by Uppal et al.^3^ We congratulate the authors for this successful publication and make some additional contributions.

In this article, 2 cases are described in which implanted percutaneous StimRouter (Bioness, Inc., Valencia, CA) peripheral nerve stimulator leads migrated and were exposed through the skin several months to years postprocedure. In both cases, concerns were raised regarding the use of traction for remote explanation, including potential for fractured and retained leads, as well as possible necessity for escalation to surgical incision and dissection. Scarring can take place at multiple points along a lead over a period of months to years, and this may be enhanced by the use of tines on the distal portion of the lead.

**Figure 1.**
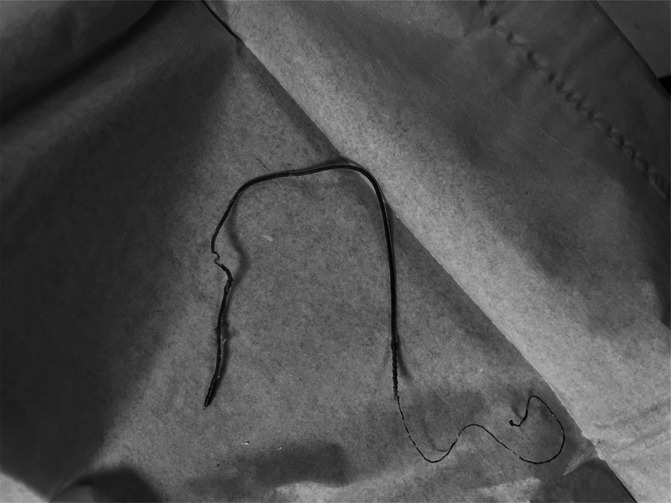
Peripheral nerve stimulator lead explanted fully intact with lead tip and hook visualized.

**Figure 2.**
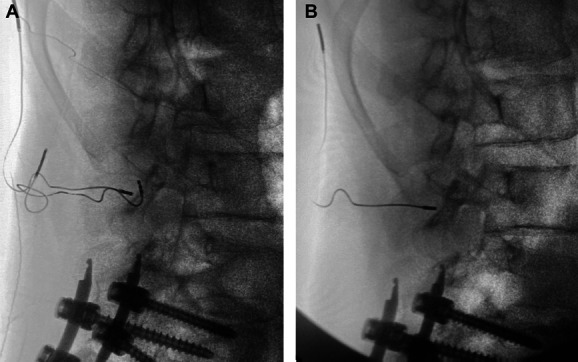
Lateral fluoroscopic images depicting: A. intact left and exposed right peripheral nerve stimulator lead to the L3 medial branches and B. successful explanation of right lead with no retained fragments.

We shared these concerns in a case similar to that of the above authors, although with a different outcome. In our case, a 51-year-old man underwent an uncomplicated permanent placement of the percutaneous StimRouter (Bioness, Inc) device targeting the L3 medial branches. Approximately 4 months after implantation, the patient returned to follow-up with the right lead protruding through the skin and loss of appropriate analgesia. Similar to the above authors, we made the decision for expedient explanation. After using simple traction under fluoroscopy, we initially encountered resistance to lead explanation. However, after infiltrating the skin and surrounding muscle tissue layers with approximately 10 ccs of 2% lidocaine, we were able to successfully explant the entire lead with traction alone, and both the lead tip and the hook were visualized fully intact (Fig. 1). Subsequent fluoroscopy was used to confirm that no lead fragments were retained (Fig. 2).

It is not exactly known how long it takes for a lead to scar into the tines or around the coil area (Fig. 2). In our case, the lead had only been indwelling for 4 months, as compared with the longer time frame of 1 and 3 years, as presented by the above authors. In such cases of peripheral nerve stimulator lead migration to the skin, we suggest a trial of simple traction, followed by intramuscular infiltration of lidocaine along the lead tract, before escalating to a surgery which could potentially be avoided. Although minimally invasive lead extraction devices are available for pacemaker leads like the one used in our VA healthcare system,^2^ these are not commonly used in pain management. Elucidating optimal time frame and technique for removal would be especially useful for the future because the MRI compatibility of these devices is limited by current FDA guidance^1^ and is a frequently cited reason for explanation.

## Disclosures

The authors have no conflicts of interest to declare.
